# Supplementary material for: Molecular Dysregulation in Autism Spectrum Disorder
Source: J Pers Med. 2021 Aug 27;11(9):848. doi: 10.3390/jpm11090848 (PMC8466026; doi:10.3390/jpm11090848)

Journal of Personalized Medicine

MDPI

Supplementary Materials:

The following supplementary materials are available online.

Supplementary Table S1: Genes associated with risk of autism spectrum disorder (ASD) [12,44,55,61,62,64,71].

Gene Symbol	Gene Name	Gene Symbol	Gene Name
ADNP	Activity Dependent Neuroprotector Homeobox	*MED13L*	Mediator complex subunit 13-like
ARID2	AT-Rich Interaction Domain 2	*MTHFR*	**Methylenetetrahydrofolate Reductase**
AMT	Aminomethyltransferase	*NCKAP1*	NCK Associated Protein 1
ANK2	Ankyrin 2	*NEGR1*	Neuronal Growth Regulator 1
BRCA2	Breast cancer 2	*NLGN3*	**Neuroligin 3**
CACNA1C	Calcium Voltage-Gated Channel Subunit Alpha1 C	*NRXN1*	Neurexin 1
CEP41	Centrosomal Protein 41	*NTNG1*	Netrin G1
CHD8	Chromodomain helicase DNA binding protein 8	*OXTR*	**Oxytocin Receptor**
CUL3	Cullin 3	*PHF3*	PHD finger protein 3
DEAF1	DEAF1 Transcription Factor	*POGZ*	Pogo Transposable Element Derived With ZNF Domain
DSCAM	DS Cell Adhesion Molecule	*PTEN*	Phosphatase and tensin homolog
FMR1	Fragile X mental retardation protein 1	*RELN*	Reelin
FOXP2	Forkhead Box P2	*SCN1A*	Sodium Voltage-Gated Channel Alpha Subunit 1
FOXP1	Forkhead Box P1	*SCN2A*	Sodium channel, voltage-gated, type II, alpha subunit
GIGYF1	GRB10 Interacting GYF Protein 1	*SETD5*	SET Domain Containing 5
GRIN2B	Glutamate Ionotropic Receptor NMDA Type Subunit 2B	*SHANK2*	SH3 and multiple ankyrin repeat domains 2
ILF2	Interleukin Enhancer Binding Factor 2	*SHANK3*	SH3 and muliple ankyrin repeat domains 3
HOXB1	Homeobox B1	*SLC6A1*	Solute Carrier Family 6 Member 1
HOXA1	Homeobox A1	*SLC6A4*	**Solute Carrier Family 6 Member 4**
HTR2A	**5-Hydroxytryptamine Receptor 2A**	*SYNE1*	Spectrin repeat containing, nuclear envelope 1

KCNMA1	Potassium large conductance calcium-activated channel, subfamily M, alpha member 1	*PHF3*	PHD finger protein 3
KCNQ3	Potassium Voltage-Gated Channel Subfamily Q Member 3	*POU3F2*	POU Class 3 Homeobox 2
KDM5B	Lysine Demethylase 5B	*POGZ*	Pogo Transposable Element Derived With ZNF Domain
KDM6B	Lysine Demethylase 6B	*POMGNT1*	Protein O-Linked Mannose N-Acetylglucosaminyltransferase 1 (Beta 1,2-)
KMT2C	Lysine Methyltransferase 2C	*PTEN*	Phosphatase and tensin homolog
KMT2E	Lysine (K)-specific methyltransferase 2E	*UBE3A*	**Ubiquitin Protein Ligase E3A**
KATNAL2	Katanin Catalytic Subunit A1 Like 2	*WDR45*	WD Repeat Domain 45
MECP2	Methyl-CpG Binding Protein 2	*WNT2*	**Wnt Family Member 2**

Supplementary Table S2: Gene ontology analysis of ASD genes from Table S1

Category	Cluster	Term	Count	%	List Total	Pop Hits	Pop Total	Fold Enrichment	pv	Bonferroni	Benjamini	FDR	Genes
GOBP	1	GO:0035176~social behavior	**8**	15	51	48	16792	54.9	9E-11	0%	0%	0%	MECP2, NLGN3, OXTR, NRXN1, PTEN, SHANK3, SHANK2, SLC6A4
GOBP	1	GO:0051968~positive regulation of synaptic transmission, glutamatergic	**6**	12	51	19	16792	104.0	2E-09	0%	0%	0%	NLGN3, OXTR, RELN, NRXN1, SHANK3, SHANK2
GOBP	1	GO:0007416~synapse assembly	**7**	13	51	61	16792	37.8	3E-08	0%	0%	0%	MECP2, NLGN3, DSCAM, NRXN1, PTEN, SHANK3, SHANK2
GOBP	1	GO:0060291~long-term synaptic potentiation	**6**	12	51	38	16792	52.0	9E-08	0%	0%	0%	MECP2, NLGN3, RELN, PTEN, SHANK3, SHANK2
GOBP	1	GO:2000463~positive regulation of excitatory postsynaptic potential	**5**	9.6	51	20	16792	82.3	3E-07	0%	0%	0%	NLGN3, RELN, NRXN1, PTEN, SHANK3
GOBP	1	GO:0030534~adult behavior	**5**	9.6	51	26	16792	63.3	1E-06	0%	0%	0%	NLGN3, NRXN1, PTEN, SHANK3, SHANK2
GOBP	1	GO:0007613~memory	**6**	12	51	62	16792	31.9	1E-06	0%	0%	0%	OXTR, PTEN, HTR2A, SHANK3, SHANK2, SLC6A4
GOBP	1	GO:0071625~vocalization behavior	**4**	7.7	51	14	16792	94.1	9E-06	1%	0%	0%	NLGN3, NRXN1, SHANK3, SHANK2
GOBP	1	GO:0007612~learning	**5**	9.6	51	57	16792	28.9	2E-05	2%	0%	0%	NLGN3, NRXN1, SLC6A1, SHANK3, SHANK2

GOBP	1	GO:2000969~positive regulation of alpha-amino-3-hydroxy-5-methyl-4-isoxazole propionate selective glutamate receptor activity	**3**	5.8	51	4	16792	246.9	5E-05	4%	0%	0%	NLGN3, RELN, SHANK3
GOBP	1	GO:0097114~NMDA glutamate receptor clustering	**3**	5.8	51	5	16792	197.6	9E-05	6%	0%	0%	RELN, NRXN1, SHANK3
GOBP	1	GO:0097105~presynaptic membrane assembly	**3**	5.8	51	9	16792	109.8	3E-04	19%	1%	1%	NLGN3, NRXN1, PTEN
GOBP	1	GO:0060292~long term synaptic depression	**3**	5.8	51	16	16792	61.7	0.001	50%	4%	4%	PTEN, SHANK3, SHANK2
GOBP	1	GO:0048854~brain morphogenesis	**3**	5.8	51	18	16792	54.9	0.001	59%	5%	5%	PTEN, SHANK3, SLC6A4
GOBP	2	GO:0007613~memory	**6**	12	51	62	16792	31.9	1E-06	0%	0%	0%	OXTR, PTEN, HTR2A, SHANK3, SHANK2, SLC6A4
GOBP	2	GO:0032355~response to estradiol	**4**	7.7	51	91	16792	14.5	0.003	82%	8%	8%	OXTR, PTEN, SLC6A1, SLC6A4
GOBP	2	GO:0042493~response to drug	**5**	9.6	51	304	16792	5.4	0.013	100%	28%	27%	OXTR, PTEN, MTHFR, HTR2A, SLC6A4
GOBP	3	GO:0034765~regulation of ion transmembrane transport	**4**	7.7	51	111	16792	11.9	0.004	95%	13%	12%	KCNMA1, KCNQ3, SCN2A, SCN1A

GOBP	5	GO:0006351~transcription, DNA-templated	**14**	27	51	1955	16792	2.4	0.004	93%	12%	11%	KMT2E, PHF3, KDM5B, KMT2C, CHD8, ILF2, FOXP2, FOXP1, MED13L, MECP2, HOXA1, ADNP, HOXB1, ARID2
GOBP	7	GO:0051968~positive regulation of synaptic transmission, glutamatergic	**6**	12	51	19	16792	104.0	2E-09	0%	0%	0%	NLGN3, OXTR, RELN, NRXN1, SHANK3, SHANK2
GOBP	7	GO:2000463~positive regulation of excitatory postsynaptic potential	**5**	9.6	51	20	16792	82.3	3E-07	0%	0%	0%	NLGN3, RELN, NRXN1, PTEN, SHANK3
GOBP	7	GO:0007268~chemical synaptic transmission	**6**	12	51	240	16792	8.2	7E-04	39%	3%	3%	NRXN1, KCNQ3, HTR2A, SLC6A1, GRIN2B, SLC6A4
GOBP	7	GO:2000310~regulation of N-methyl-D-aspartate selective glutamate receptor activity	**3**	5.8	51	15	16792	65.9	9E-04	46%	4%	4%	NLGN3, RELN, NRXN1
GOBP	8	GO:0045892~negative regulation of transcription, DNA-templated	**6**	12	51	499	16792	4.0	0.016	100%	30%	29%	MECP2, KDM5B, DEAF1, CHD8, FOXP2, FOXP1
GOBP	8	GO:0009791~post-embryonic development	**3**	5.8	51	73	16792	13.5	0.02	100%	31%	30%	MECP2, KDM5B, FOXP2

GOBP	8	GO:0000122~negative regulation of transcription from RNA polymerase II promoter	**5**	9.6	51	720	16792	2.3	0.166	100%	100%	97%	MECP2, CHD8, CUL3, FOXP2, FOXP1
GOBP	9	GO:0006355~regulation of transcription, DNA-templated	**5**	9.6	51	1504	16792	1.1	0.666	100%	100%	97%	KMT2C, HOXA1, ADNP, HOXB1, ARID2
GOCC	1	GO:0043005~neuron projection	**8**	15	49	237	18224	12.6	3E-06	0%	0%	0%	FMR1, PTEN, ANK2, SLC6A1, GRIN2B, SHANK3, SHANK2, SLC6A4
GOCC	1	GO:0045211~postsynaptic membrane	**7**	13	49	211	18224	12.3	2E-05	0%	0%	0%	FMR1, PTEN, ANK2, GRIN2B, SHANK3, SHANK2, SYNE1
GOCC	1	GO:0030054~cell junction	**7**	13	49	459	18224	5.7	0.001	16%	4%	3%	NLGN3, DSCAM, NRXN1, FMR1, GRIN2B, SHANK3, SHANK2
GOCC	1	GO:0043197~dendritic spine	**4**	7.7	49	100	18224	14.9	0.002	28%	4%	4%	FMR1, PTEN, SHANK3, SHANK2
GOCC	1	GO:0014069~postsynaptic density	**4**	7.7	49	184	18224	8.1	0.013	83%	13%	12%	FMR1, CACNA1C, SHANK3, SHANK2
GOCC	1	GO:0030426~growth cone	**3**	5.8	49	116	18224	9.6	0.037	100%	27%	25%	DSCAM, FMR1, SHANK2
GOCC	1	GO:0005622~intracellular	**4**	7.7	49	1332	18224	1.1	0.692	100%	100%	93%	OXTR, ANK2, GRIN2B, SHANK3
GOCC	3	GO:0033268~node of Ranvier	**3**	5.8	49	15	18224	74.4	7E-04	9%	2%	2%	KCNQ3, SCN2A, SCN1A
GOCC	5	GO:0005654~nucleoplasm	**15**	29	49	2784	18224	2.0	0.011	78%	13%	12%	KMT2E, KDM6B, KDM5B, CUL3, KMT2C, CHD8, FMR1, PTEN, BRCA2,

													ILF2, FOXP1, SYNE1, DEAF1, POGZ, ARID2
GOCC	5	GO:0005634~nucleus	**21**	40	49	5415	18224	1.4	0.052	100%	35%	32%	KDM6B, KDM5B, CUL3, KMT2C, CHD8, FMR1, PTEN, UBE3A, BRCA2, POU3F2, ILF2, FOXP2, FOXP1, SYNE1, MECP2, KATNAL2, DEAF1, POGZ, HOXA1, ADNP, HOXB1
GOCC	7	GO:0005886~plasma membrane	**21**	40	49	4121	18224	1.9	0.002	29%	4%	4%	KMT2E, OXTR, DSCAM, NEGR1, NRXN1, PTEN, ANK2, CACNA1C, HTR2A, SLC6A1, GRIN2B, SLC6A4, RELN, KCNMA1, KCNQ3, CEP41, ARID2, SHANK3, WNT2, SHANK2, SCN1A
GOCC	7	GO:0005887~integral component of plasma membrane	**9**	17	49	1415	18224	2.4	0.03	99%	25%	23%	NLGN3, OXTR, DSCAM, NRXN1, HTR2A, SLC6A1, SCN2A, GRIN2B, SLC6A4
GOCC	7	GO:0016021~integral component of membrane	**14**	27	49	5163	18224	1.0	0.63	100%	100%	93%	NLGN3, NCKAP1, OXTR, NRXN1, CACNA1C, SLC6A1, BRCA2, WDR45, SLC6A4, SYNE1, KCNMA1, SCN2A, SHANK2, SCN1A
GOCC	10	GO:0005576~extracellular region	**4**	7.7	49	1610	18224	0.9	0.809	100%	100%	93%	DSCAM, DEAF1, PTEN, WNT2
GOMF	4	GO:0046872~metal ion binding	**14**	27	50	2069	16881	2.3	0.005	59%	32%	32%	KDM6B, NRXN1, UBE3A, CACNA1C, SLC6A1, FOXP2, SLC6A4, FOXP1, RELN, DEAF1, KCNMA1, POGZ, ADNP, ARID2

GOMF	5	GO:0003677~DNA binding	**11**	21	50	1674	16881	2.2	0.02	97%	68%	68%	MECP2, KDM5B, DEAF1, CHD8, KMT2C, POGZ, ADNP, HOXB1, ARID2, ILF2, FOXP2
GOMF	8	GO:0003700~transcription factor activity, sequence-specific DNA binding	**6**	12	50	961	16881	2.1	0.145	100%	100%	100%	MECP2, KDM5B, DEAF1, POU3F2, FOXP2, FOXP1
Pathways	1	hsa04724:Glutamatergic synapse	**4**	7.7	30	114	6879	8.0	0.012	62%	87%	87%	CACNA1C, GRIN2B, SHANK3, SHANK2
Pathways	3	R-HSA-445095:R-HSA-445095	**4**	7.7	33	31	9075	35.5	2E-04	1%	1%	1%	KCNQ3, ANK2, SCN2A, SCN1A
Pathways	3	R-HSA-5576892:R-HSA-5576892	**3**	5.8	33	46	9075	17.9	0.011	51%	35%	35%	CACNA1C, SCN2A, SCN1A
Pathways	3	hsa04728:Dopaminergic synapse	**3**	5.8	30	128	6879	5.4	0.101	100%	100%	100%	CACNA1C, GRIN2B, SCN1A
Pathways	7	hsa04514:Cell adhesion molecules (CAMs)	**4**	7.7	30	142	6879	6.5	0.021	83%	87%	87%	NTNG1, NLGN3, NEGR1, NRXN1
Keywords	1	Synapse	**8**	15	52	357	20581	8.9	3E-05	0%	0%	0%	NLGN3, DSCAM, NRXN1, FMR1, ANK2, GRIN2B, SHANK3, SHANK2
Keywords	1	Postsynaptic cell membrane	**5**	9.6	52	179	20581	11.1	0.001	14%	1%	1%	FMR1, ANK2, GRIN2B, SHANK3, SHANK2
Keywords	1	Cell junction	**8**	15	52	675	20581	4.7	0.001	18%	1%	1%	NLGN3, DSCAM, NRXN1, FMR1, ANK2, GRIN2B, SHANK3, SHANK2
Keywords	1	Cell projection	**7**	13	52	721	20581	3.8	0.009	72%	5%	4%	NCKAP1, DSCAM, FMR1, CEP41, HTR2A, SHANK3, SHANK2

Key-words	1	ANK repeat	**3**	5.8	52	264	20581	4.5	0.139	100%	48%	40%	ANK2, SHANK3, SHANK2
Key-words	3	Epilepsy	**7**	13	52	127	20581	21.8	7E-07	0%	0%	0%	RELN, KCNMA1, KCNQ3, SLC6A1, SCN2A, GRIN2B, SCN1A
Key-words	3	Voltage-gated channel	**5**	9.6	52	150	20581	13.2	5E-04	7%	1%	1%	KCNMA1, KCNQ3, CACNA1C, SCN2A, SCN1A
Key-words	3	Ion channel	**6**	12	52	359	20581	6.6	0.002	25%	2%	1%	KCNMA1, KCNQ3, CACNA1C, SCN2A, GRIN2B, SCN1A
Key-words	3	Ion transport	**6**	12	52	642	20581	3.7	0.021	96%	11%	9%	KCNMA1, KCNQ3, CACNA1C, SCN2A, GRIN2B, SCN1A
Key-words	3	Transport	**11**	21	52	1978	20581	2.2	0.022	96%	11%	9%	CUL3, FMR1, KCNMA1, KCNQ3, CEP41, CACNA1C, SLC6A1, SCN2A, GRIN2B, SCN1A, SLC6A4
Key-words	4	Metal-binding	**19**	37	52	3640	20581	2.1	0.002	26%	2%	1%	KMT2E, PHF3, KDM6B, KDM5B, NRXN1, KMT2C, UBE3A, CACNA1C, SLC6A1, GRIN2B, FOXP2, SLC6A4, FOXP1, RELN, DEAF1, KCNMA1, POGZ, ADNP, ARID2
Key-words	4	Zinc	**14**	27	52	2348	20581	2.4	0.004	44%	3%	2%	KMT2E, PHF3, KDM6B, KDM5B, KMT2C, UBE3A, GRIN2B, FOXP2, FOXP1, RELN, DEAF1, POGZ, ADNP, ARID2
Key-words	4	Zinc-finger	**11**	21	52	1781	20581	2.4	0.011	81%	6%	5%	KMT2E, PHF3, KDM5B, DEAF1, KMT2C, POGZ, UBE3A, ADNP, ARID2, FOXP2, FOXP1
Key-words	5	DNA-binding	**14**	27	52	2050	20581	2.7	0.001	16%	1%	1%	KMT2C, CHD8, BRCA2, POU3F2, ILF2, FOXP2, FOXP1, MECP2, DEAF1,

													POGZ, HOXA1, ADNP, HOXB1, ARID2
Key-words	5	Transcription regulation	**15**	29	52	2332	20581	2.5	0.001	17%	1%	1%	KMT2E, KDM5B, KMT2C, CHD8, POU3F2, ILF2, FOXP2, FOXP1, MED13L, MECP2, DEAF1, HOXA1, ADNP, HOXB1, ARID2
Key-words	5	Transcription	**15**	29	52	2398	20581	2.5	0.002	21%	1%	1%	KMT2E, KDM5B, KMT2C, CHD8, POU3F2, ILF2, FOXP2, FOXP1, MED13L, MECP2, DEAF1, HOXA1, ADNP, HOXB1, ARID2
Key-words	5	Nucleus	**23**	44	52	5244	20581	1.7	0.004	49%	3%	2%	KMT2E, KDM6B, KDM5B, CUL3, KMT2C, CHD8, FMR1, PTEN, UBE3A, BRCA2, POU3F2, ILF2, FOXP2, FOXP1, SYNE1, MED13L, MECP2, DEAF1, POGZ, HOXA1, ADNP, HOXB1, ARID2
Key-words	6	Chromosomal rearrangement	**6**	12	52	334	20581	7.1	0.001	19%	1%	1%	MECP2, GRIN2B, SHANK3, FOXP2, MED13L, FOXP1
Key-words	6	Repressor	**7**	13	52	592	20581	4.7	0.003	39%	2%	2%	MECP2, KDM5B, CHD8, FMR1, FOXP2, MED13L, FOXP1
Key-words	7	Cell membrane	**20**	38	52	3175	20581	2.5	1E-04	2%	0%	0%	KMT2E, NTNG1, NLGN3, NCKAP1, OXTR, DSCAM, NEGR1, NRXN1, FMR1, ANK2, CACNA1C, HTR2A, SLC6A1, GRIN2B, SLC6A4, KCNMA1, SCN2A, SHANK3, SHANK2, SCN1A
Key-words	7	Cell adhesion	**5**	9.6	52	479	20581	4.1	0.031	99%	14%	12%	NLGN3, RELN, DSCAM, NEGR1, NRXN1

Key-words	7	Disulfide bond	**14**	27	52	3434	20581	1.6	0.072	100%	30%	25%	NTNG1, NLGN3, OXTR, DSCAM, NEGR1, NRXN1, CACNA1C, HTR2A, SLC6A1, GRIN2B, SLC6A4, RELN, SCN2A, WNT2
Key-words	7	Glycoprotein	**17**	33	52	4551	20581	1.5	0.081	100%	32%	27%	KMT2E, NTNG1, NLGN3, OXTR, DSCAM, NEGR1, NRXN1, CACNA1C, HTR2A, SLC6A1, GRIN2B, SLC6A4, RELN, SCN2A, WNT2, SHANK2, SCN1A
Key-words	7	Membrane	**24**	46	52	7494	20581	1.3	0.127	100%	45%	38%	KMT2E, NTNG1, NLGN3, NCKAP1, OXTR, DSCAM, NEGR1, NRXN1, FMR1, ANK2, CACNA1C, HTR2A, SLC6A1, BRCA2, WDR45, GRIN2B, SLC6A4, SYNE1, KCNMA1, KCNQ3, SCN2A, SHANK3, SHANK2, SCN1A
Key-words	7	Transmembrane helix	**18**	35	52	5634	20581	1.3	0.21	100%	60%	50%	NLGN3, NCKAP1, OXTR, DSCAM, NRXN1, CACNA1C, HTR2A, SLC6A1, BRCA2, WDR45, GRIN2B, SLC6A4, SYNE1, KCNMA1, KCNQ3, SCN2A, SHANK2, SCN1A
Key-words	7	Transmembrane	**18**	35	52	5651	20581	1.3	0.214	100%	60%	50%	NLGN3, NCKAP1, OXTR, DSCAM, NRXN1, CACNA1C, HTR2A, SLC6A1, BRCA2, WDR45, GRIN2B, SLC6A4, SYNE1, KCNMA1, KCNQ3, SCN2A, SHANK2, SCN1A

Key-words	7	Signal	**11**	21	52	4160	20581	1.0	0.598	100%	100%	84%	NTNG1, NLGN3, RELN, DSCAM, NEGR1, NRXN1, KCNQ3, SCN2A, WNT2, GRIN2B, SLC6A4
Key-words	9	Homeobox	**4**	7.7	52	262	20581	6.0	0.027	98%	13%	11%	HOXA1, ADNP, HOXB1, POU3F2
Key-words	10	Neurogenesis	**5**	9.6	52	250	20581	7.9	0.003	40%	2%	2%	NTNG1, DSCAM, DEAF1, FMR1, PTEN
Key-words	10	Secreted	**5**	9.6	52	1965	20581	1.0	0.73	100%	100%	84%	RELN, DSCAM, DEAF1, PTEN, WNT2
Key-words	11	Ubl conjugation	**8**	15	52	1705	20581	1.9	0.126	100%	45%	38%	KDM5B, CHD8, CUL3, FMR1, PTEN, ADNP, BRCA2, SCN2A
Key-words	11	Isopeptide bond	**5**	9.6	52	1132	20581	1.7	0.308	100%	74%	62%	KDM5B, CHD8, CUL3, PTEN, ADNP
Key-words	11	Acetylation	**9**	17	52	3424	20581	1.0	0.63	100%	100%	84%	MECP2, NCKAP1, KDM5B, CUL3, KMT2C, FMR1, PTEN, ADNP, ARID2
Feature	3	repeat:I	**3**	5.8	51	26	20063	45.4	0.002	47%	11%	11%	CACNA1C, SCN2A, SCN1A
Feature	3	repeat:II	**3**	5.8	51	26	20063	45.4	0.002	47%	11%	11%	CACNA1C, SCN2A, SCN1A
Feature	3	repeat:III	**3**	5.8	51	26	20063	45.4	0.002	47%	11%	11%	CACNA1C, SCN2A, SCN1A
Feature	3	repeat:IV	**3**	5.8	51	26	20063	45.4	0.002	47%	11%	11%	CACNA1C, SCN2A, SCN1A
Feature	6	domain:Leucine-zipper	**3**	5.8	51	113	20063	10.4	0.032	100%	68%	67%	FOXP2, MED13L, FOXP1
Feature	7	glycosylation site:N-linked (GlcNAc...)	**15**	29	51	4234	20063	1.4	0.153	100%	100%	99%	NTNG1, NLGN3, OXTR, DSCAM, NEGR1, NRXN1, CACNA1C, HTR2A, SLC6A1, GRIN2B, SLC6A4, RELN, SCN2A, WNT2, SCN1A

Feature	7	topological domain:Extracellular	**10**	19	51	2787	20063	1.4	0.253	100%	100%	99%	NLGN3, OXTR, DSCAM, NRXN1, KCNMA1, CACNA1C, HTR2A, SLC6A1, GRIN2B, SLC6A4
Feature	7	topological domain:Cytoplasmic	**11**	21	51	3456	20063	1.3	0.356	100%	100%	99%	NLGN3, OXTR, DSCAM, NRXN1, KCNMA1, CACNA1C, HTR2A, SLC6A1, GRIN2B, SYNE1, SLC6A4
Feature	7	transmembrane region	**15**	29	51	5056	20063	1.2	0.375	100%	100%	99%	NLGN3, NCKAP1, OXTR, DSCAM, NRXN1, CACNA1C, HTR2A, SLC6A1, GRIN2B, SLC6A4, SYNE1, KCNMA1, KCNQ3, SCN2A, SCN1A
Feature	7	disulfide bond	**8**	15	51	2917	20063	1.1	0.604	100%	100%	99%	NTNG1, NLGN3, OXTR, RELN, DSCAM, NEGR1, NRXN1, HTR2A
Feature	7	signal peptide	**8**	15	51	3346	20063	0.9	0.75	100%	100%	99%	NTNG1, NLGN3, RELN, DSCAM, NEGR1, NRXN1, WNT2, GRIN2B
Feature	9	DNA-binding region:Homeobox	**4**	7.7	51	191	20063	8.2	0.012	98%	34%	33%	HOXA1, ADNP, HOXB1, POU3F2

**Category**	**Term**	**Count**	**%**	**List Total**	**Pop Hits**	**Pop Total**	**Fold**	**pv**	**Bonferroni**	**Benjamini**	**FDR**	**Genes**
GOBP	GO:0051965~positive regulation of synapse assembly	**5**	10	51	62	16792	26.6	3E-05	2%	0%	0%	MECP2, NLGN3, OXTR, NRXN1, ADNP
GOBP	GO:0042297~vocal learning	**3**	6	51	7	16792	141.1	2E-04	12%	1%	1%	NRXN1, SHANK3, FOXP2
GOBP	GO:0050795~regulation of behavior	**3**	6	51	9	16792	109.8	3E-04	19%	1%	1%	RELN, HOXA1, HTR2A

GOBP	GO:0007626~locomotory behavior	**4**	8	51	84	16792	15.7	0.002	75%	7%	7%	MECP2, DSCAM, NEGR1, PTEN
GOBP	GO:0002053~positive regulation of mesenchymal cell proliferation	**3**	6	51	26	16792	38.0	0.003	84%	8%	8%	WNT2, FOXP2, FOXP1
GOBP	GO:0008542~visual learning	**3**	6	51	45	16792	22.0	0.008	100%	20%	20%	MECP2, NLGN3, DEAF1
GOBP	GO:0008284~positive regulation of cell proliferation	**6**	12	51	466	16792	4.2	0.012	100%	28%	27%	MECP2, CUL3, PTEN, HTR2A, WNT2, POU3F2
GOBP	GO:0001666~response to hypoxia	**4**	8	51	172	16792	7.7	0.015	100%	29%	28%	MECP2, KCNMA1, MTHFR, SLC6A4
GOBP	GO:0045893~positive regulation of transcription, DNA-templated	**6**	12	51	515	16792	3.8	0.018	100%	30%	29%	KMT2E, MECP2, DEAF1, CHD8, BRCA2, ILF2
GOBP	GO:0071300~cellular response to retinoic acid	**3**	6	51	70	16792	14.1	0.018	100%	30%	29%	KMT2E, WNT2, SLC6A4
GOBP	GO:0007420~brain development	**4**	8	51	190	16792	6.9	0.019	100%	30%	29%	RELN, CHD8, UBE3A, BRCA2
GOBP	GO:0050731~positive regulation of peptidyl-tyrosine phosphorylation	**3**	6	51	82	16792	12.0	0.025	100%	35%	34%	RELN, ADNP, HTR2A

GOBP	GO:0060070~canonical Wnt signaling pathway	**3**	6	51	83	16792	11.9	0.025	100%	35%	34%	CHD8, PTEN, WNT2
GOBP	GO:0007417~central nervous system development	**3**	6	51	120	16792	8.2	0.05	100%	50%	49%	NCKAP1, RELN, PTEN
GOBP	GO:0045944~positive regulation of transcription from RNA polymerase II promoter	**7**	13	51	981	16792	2.3	0.07	100%	66%	64%	KDM6B, CHD8, UBE3A, HOXB1, WNT2, POU3F2, FOXP1
GOBP	GO:0007507~heart development	**3**	6	51	183	16792	5.4	0.103	100%	83%	80%	OXTR, PTEN, CACNA1C
GOBP	GO:0010628~positive regulation of gene expression	**3**	6	51	262	16792	3.8	0.183	100%	100%	97%	KDM5B, ANK2, SLC6A4
GOBP	GO:0000165~MAPK cascade	**3**	6	51	262	16792	3.8	0.183	100%	100%	97%	CUL3, GRIN2B, SHANK3
GOBP	GO:0007275~multicellular organism development	**4**	8	51	521	16792	2.5	0.202	100%	100%	97%	PHF3, HOXA1, HOXB1, WNT2
GOBP	GO:0006357~regulation of transcription from RNA polymerase II promoter	**3**	6	51	441	16792	2.2	0.379	100%	100%	97%	DEAF1, MED13L, FOXP1

GOBP	GO:0055114~oxidation-reduction process	**3**	6	51	592	16792	1.7	0.53	100%	100%	97%	KDM6B, KDM5B, MTHFR
GOCC	GO:0030424~axon	**6**	12	49	222	18224	10.1	3E-04	4%	1%	1%	DSCAM, FMR1, ADNP, HTR2A, SLC6A1, SCN2A
GOCC	GO:0098794~postsynapse	**3**	6	49	22	18224	50.7	0.002	19%	4%	3%	MECP2, NLGN3, FMR1
GOCC	GO:0016324~apical plasma membrane	**5**	10	49	291	18224	6.4	0.007	64%	11%	10%	OXTR, KCNMA1, PTEN, ANK2, SHANK2
GOCC	GO:0043025~neuronal cell body	**5**	10	49	315	18224	5.9	0.009	74%	13%	12%	NRXN1, ADNP, HTR2A, SHANK2, SCN1A
GOCC	GO:0045202~synapse	**4**	8	49	181	18224	8.2	0.012	82%	13%	12%	NLGN3, DSCAM, FMR1, MTHFR
GOCC	GO:0098793~presynapse	**3**	6	49	64	18224	17.4	0.012	83%	13%	12%	NRXN1, FMR1, SLC6A4
GOCC	GO:0009986~cell surface	**6**	12	49	542	18224	4.1	0.014	86%	13%	12%	NLGN3, NRXN1, KCNQ3, CEP41, SLC6A1, GRIN2B
GOCC	GO:0030018~Z disc	**3**	6	49	118	18224	9.5	0.039	100%	27%	25%	ANK2, CACNA1C, SCN1A
GOCC	GO:0030425~dendrite	**4**	8	49	335	18224	4.4	0.058	100%	37%	35%	RELN, FMR1, ADNP, HTR2A
GOCC	GO:0005737~cytoplasm	**17**	33	49	###	18224	1.2	0.283	100%	100%	93%	KMT2E, KDM5B, FMR1, PTEN, UBE3A, CACNA1C, BRCA2, ILF2, SYNE1, RELN, KATNAL2, DEAF1, POGZ, ADNP, SHANK3, WNT2, SHANK2
GOCC	GO:0043234~protein complex	**3**	6	49	412	18224	2.7	0.296	100%	100%	93%	CHD8, NRXN1, BRCA2
GOCC	GO:0005829~cytosol	**11**	21	49	###	18224	1.2	0.373	100%	100%	93%	MECP2, NCKAP1, CUL3, PTEN, MTHFR, UBE3A, ANK2, CEP41, HTR2A, WDR45, SLC6A4
GOCC	GO:0005615~extracellular space	**5**	10	49	###	18224	1.4	0.478	100%	100%	93%	MECP2, NLGN3, RELN, ADNP, WNT2

GOCC	GO:0016020~membrane	**7**	13	49	###	18224	1.2	0.529	100%	100%	93%	DSCAM, CUL3, FMR1, KCNMA1, CEP41, SLC6A1, ILF2
GOCC	GO:0005730~nucleolus	**3**	6	49	857	18224	1.3	0.667	100%	100%	93%	DEAF1, FMR1, ILF2
GOCC	GO:0005739~mitochondrion	**3**	6	49	###	18224	0.8	0.875	100%	100%	93%	MECP2, PTEN, AMT
GOCC	GO:0070062~extracellular exosome	**4**	8	49	###	18224	0.5	0.985	100%	100%	98%	NCKAP1, NEGR1, CUL3, KCNMA1
GOMF	GO:0005515~protein binding	**36**	69	50	###	16881	1.4	0.004	55%	32%	32%	NLGN3, KMT2E, KDM5B, NCKAP1, CUL3, KMT2C, CHD8, FMR1, NRXN1, PTEN, UBE3A, CACNA1C, BRCA2, SLC6A4, SYNE1, MECP2, DEAF1, POGZ, HOXA1, ARID2, WNT2, NTNG1, KDM6B, DSCAM, NEGR1, ANK2, POU3F2, ILF2, GRIN2B, FOXP2, FOXP1, KCNMA1, CEP41, ADNP, SHANK3, SHANK2
GOMF	GO:0003682~chromatin binding	**6**	12	50	391	16881	5.2	0.005	61%	32%	32%	MECP2, KDM6B, CHD8, FMR1, ADNP, FOXP1
GOMF	GO:0043565~sequence-specific DNA binding	**5**	10	50	518	16881	3.3	0.063	100%	95%	95%	KDM6B, HOXA1, HOXB1, FOXP2, FOXP1
GOMF	GO:0032403~protein complex binding	**3**	6	50	206	16881	4.9	0.12	100%	100%	100%	NCKAP1, MTHFR, HTR2A
GOMF	GO:0042803~protein homodimerization activity	**5**	10	50	730	16881	2.3	0.161	100%	100%	100%	CUL3, FMR1, FOXP2, SYNE1, SLC6A4
GOMF	GO:0003779~actin binding	**3**	6	50	278	16881	3.6	0.193	100%	100%	100%	KCNMA1, SHANK3, SYNE1

GOMF	GO:0008270~zinc ion binding	**6**	12	50	###	16881	1.7	0.25	100%	100%	100%	KMT2E, PHF3, KDM5B, KMT2C, GRIN2B, SHANK3
GOMF	GO:0019899~enzyme binding	**3**	6	50	333	16881	3.0	0.252	100%	100%	100%	KMT2E, PTEN, ANK2
GOMF	GO:0000978~RNA polymerase II core promoter proximal region sequence-specific DNA binding	**3**	6	50	355	16881	2.9	0.276	100%	100%	100%	POU3F2, FOXP2, FOXP1
GOMF	GO:0042802~identical protein binding	**4**	8	50	749	16881	1.8	0.371	100%	100%	100%	FMR1, PTEN, HOXA1, POU3F2
GOMF	GO:0046982~protein heterodimerization activity	**3**	6	50	465	16881	2.2	0.393	100%	100%	100%	CUL3, FMR1, FOXP2
GOMF	GO:0044822~poly(A) RNA binding	**5**	10	50	###	16881	1.5	0.417	100%	100%	100%	MECP2, KMT2C, FMR1, ILF2, SYNE1
GOMF	GO:0005524~ATP binding	**3**	6	50	###	16881	0.7	0.939	100%	100%	100%	KATNAL2, CHD8, ILF2
Pathway	R-HSA-5617472:R-HSA-5617472	**3**	6	33	122	9075	6.8	0.068	99%	100%	100%	KMT2C, HOXA1, HOXB1
Pathway	hsa04726:Serotonergic synapse	**3**	6	30	111	6879	6.2	0.079	100%	100%	100%	CACNA1C, HTR2A, SLC6A4
Pathway	hsa04020:Calcium signaling pathway	**3**	6	30	179	6879	3.8	0.174	100%	100%	100%	OXTR, CACNA1C, HTR2A
Pathway	hsa04024:cAMP signaling pathway	**3**	6	30	198	6879	3.5	0.203	100%	100%	100%	OXTR, CACNA1C, GRIN2B

Pathway	hsa04080:Neuroactive ligand-receptor interaction	**3**	6	30	277	6879	2.5	0.327	100%	100%	100%	OXTR, HTR2A, GRIN2B
Pathway	hsa05200:Pathways in cancer	**3**	6	30	393	6879	1.8	0.5	100%	100%	100%	PTEN, BRCA2, WNT2
Keywords	Autism spectrum disorder	**13**	25	52	40	20581	128.6	7E-23	0%	0%	0%	NLGN3, SETD5, CHD8, PTEN, CACNA1C, MECP2, POGZ, HOXA1, CEP41, ADNP, SHANK3, SHANK2, SCN1A
Keywords	Autism	**8**	15	52	25	20581	126.7	2E-13	0%	0%	0%	MECP2, NLGN3, SETD5, CHD8, CEP41, CACNA1C, SHANK2, SCN1A
Keywords	Disease mutation	**27**	52	52	###	20581	4.2	3E-11	0%	0%	0%	NLGN3, CUL3, CHD8, FMR1, PTEN, UBE3A, CACNA1C, SLC6A1, BRCA2, SYNE1, MECP2, RELN, DEAF1, POGZ, SCN1A, AMT, MTHFR, ANK2, GRIN2B, FOXP2, FOXP1, MED13L, KCNMA1, KCNQ3, HOXB1, SCN2A, SHANK3
Keywords	Phosphoprotein	**40**	77	52	###	20581	1.9	1E-07	0%	0%	0%	NLGN3, KMT2E, PHF3, KDM5B, SETD5, OXTR, CUL3, KMT2C, CHD8, FMR1, PTEN, UBE3A, CACNA1C, HTR2A, SLC6A1, BRCA2, SLC6A4, SYNE1, MECP2, DEAF1, POGZ, ARID2, SCN1A, KDM6B, DSCAM, NEGR1, ANK2, POU3F2, ILF2, GRIN2B, FOXP1, MED13L, GIGYF1, KCNMA1, KCNQ3, CEP41, ADNP, SCN2A, SHANK3, SHANK2
Keywords	Mental retardation	**9**	17	52	299	20581	11.9	7E-07	0%	0%	0%	MECP2, SETD5, DEAF1, FMR1, POGZ, ADNP, GRIN2B, MED13L, FOXP1
Keywords	Alternative splicing	**41**	79	52	###	20581	1.5	6E-05	1%	0%	0%	NLGN3, KMT2E, PHF3, KDM5B, NCKAP1, SETD5, CUL3, KMT2C, CHD8, FMR1, NRXN1, PTEN, UBE3A, CACNA1C, HTR2A, WDR45, SLC6A4, SYNE1, MECP2,

												RELN, DEAF1, POGZ, HOXA1, ARID2, SCN1A, NTNG1, KDM6B, DSCAM, NEGR1, AMT, MTHFR, ANK2, FOXP2, FOXP1, KATNAL2, KCNMA1, KCNQ3, CEP41, HOXB1, SCN2A, SHANK2
Key-words	Chromatin regulator	**6**	12	52	287	20581	8.3	7E-04	10%	1%	1%	KMT2E, KDM6B, KDM5B, CHD8, KMT2C, ARID2
Key-words	Sodium	**4**	8	52	124	20581	12.8	0.004	42%	2%	2%	SLC6A1, SCN2A, SCN1A, SLC6A4
Key-words	Methylation	**8**	15	52	###	20581	3.2	0.011	82%	6%	5%	MECP2, PHF3, KMT2C, FMR1, CEP41, ADNP, ILF2, SHANK3
Key-words	Activator	**6**	12	52	661	20581	3.6	0.023	97%	12%	10%	CHD8, KMT2C, FMR1, ILF2, POU3F2, MED13L
Key-words	Host-virus interaction	**4**	8	52	385	20581	4.1	0.07	100%	30%	25%	NCKAP1, FMR1, UBE3A, HTR2A
Key-words	Methyltransferase	**3**	6	52	193	20581	6.2	0.083	100%	32%	27%	KMT2E, KMT2C, AMT
Key-words	Developmental protein	**6**	12	52	949	20581	2.5	0.085	100%	32%	27%	NTNG1, RELN, DEAF1, HOXA1, HOXB1, WNT2
Key-words	Polymorphism	**35**	67	52	###	20581	1.2	0.149	100%	49%	41%	KMT2E, PHF3, SETD5, OXTR, CUL3, KMT2C, FMR1, NRXN1, PTEN, UBE3A, CACNA1C, HTR2A, SLC6A1, BRCA2, SLC6A4, SYNE1, MECP2, RELN, DEAF1, POGZ, HOXA1, WNT2, SCN1A, KDM6B, DSCAM, MTHFR, ANK2, GRIN2B, FOXP1, KATNAL2, KCNQ3, CEP41, HOXB1, SCN2A, SHANK3
Key-words	Lipoprotein	**5**	10	52	852	20581	2.3	0.16	100%	52%	43%	NTNG1, NEGR1, KMT2C, KCNMA1, WNT2

Key-words	Calcium	**5**	10	52	877	20581	2.3	0.172	100%	53%	45%	RELN, NRXN1, KCNMA1, CACNA1C, GRIN2B
Key-words	Coiled coil	**11**	21	52	###	20581	1.4	0.212	100%	60%	50%	KMT2E, GIGYF1, SETD5, KMT2C, POGZ, CACNA1C, SCN2A, SHANK3, FOXP2, SYNE1, SCN1A
Key-words	Cytoskeleton	**5**	10	52	###	20581	1.7	0.311	100%	74%	62%	KATNAL2, ANK2, CEP41, BRCA2, SYNE1
Key-words	Cytoplasm	**14**	27	52	###	20581	1.2	0.415	100%	92%	77%	KMT2E, FMR1, PTEN, UBE3A, ANK2, BRCA2, ILF2, SYNE1, KATNAL2, DEAF1, POGZ, CEP41, SHANK3, SHANK2
Key-words	Oxidoreductase	**3**	6	52	582	20581	2.0	0.425	100%	93%	78%	KDM6B, KDM5B, MTHFR
Key-words	Cell cycle	**3**	6	52	650	20581	1.8	0.482	100%	100%	84%	KMT2E, POGZ, BRCA2
Key-words	Hydrolase	**4**	8	52	###	20581	0.9	0.794	100%	100%	84%	RELN, KATNAL2, CHD8, PTEN
Key-words	Transferase	**4**	8	52	###	20581	0.9	0.807	100%	100%	84%	KMT2E, KMT2C, AMT, UBE3A
Key-words	Receptor	**3**	6	52	###	20581	0.7	0.923	100%	100%	92%	OXTR, HTR2A, GRIN2B
Feature	splice variant	**34**	65	51	###	20063	1.7	8E-05	3%	3%	3%	NLGN3, KMT2E, PHF3, KDM5B, NCKAP1, SETD5, CUL3, KMT2C, CHD8, FMR1, NRXN1, UBE3A, CACNA1C, WDR45, SYNE1, MECP2, RELN, DEAF1, POGZ, HOXA1, ARID2, SCN1A, NTNG1, DSCAM, ANK2, POU3F2, FOXP2, FOXP1, KATNAL2, KCNMA1, CEP41, SCN2A, SHANK3, SHANK2
Feature	compositionally biased region:Gln-rich	**5**	10	51	152	20063	12.9	6E-04	17%	9%	9%	GIGYF1, CHD8, KMT2C, ARID2, FOXP1

Feature	compositionally biased region:Poly-Gly	**5**	10	51	292	20063	6.7	0.006	87%	22%	22%	GIGYF1, KCNMA1, KCNQ3, CACNA1C, POU3F2
Feature	zinc finger region:C2H2-type	**3**	6	51	49	20063	24.1	0.007	89%	22%	22%	ARID2, FOXP2, FOXP1
Feature	domain:SET	**3**	6	51	49	20063	24.1	0.007	89%	22%	22%	KMT2E, SETD5, KMT2C
Feature	compositionally biased region:Pro-rich	**8**	15	51	978	20063	3.2	0.01	97%	31%	31%	KMT2E, MECP2, PHF3, DEAF1, KMT2C, POGZ, SHANK3, SHANK2
Feature	short sequence motif:PDZ-binding	**3**	6	51	77	20063	15.3	0.016	100%	41%	41%	HTR2A, SLC6A1, GRIN2B
Feature	mutagenesis site	**12**	23	51	###	20063	2.2	0.017	100%	42%	41%	KMT2E, KDM5B, DEAF1, CHD8, FMR1, KCNMA1, PTEN, KCNQ3, ANK2, CACNA1C, HTR2A, SYNE1
Feature	compositionally biased region:Poly-Gln	**3**	6	51	156	20063	7.6	0.058	100%	100%	99%	GIGYF1, POU3F2, FOXP2
Feature	sequence variant	**37**	71	51	###	20063	1.2	0.093	100%	100%	99%	NLGN3, KMT2E, PHF3, SETD5, OXTR, CUL3, KMT2C, FMR1, NRXN1, PTEN, UBE3A, CACNA1C, HTR2A, SLC6A1, BRCA2, SLC6A4, SYNE1, MECP2, RELN, DEAF1, POGZ, HOXA1, WNT2, SCN1A, DSCAM, AMT, MTHFR, ANK2, GRIN2B, FOXP2, MED13L, KATNAL2, KCNMA1, KCNQ3, HOXB1, SCN2A, SHANK3
Feature	compositionally biased region:Ser-rich	**4**	8	51	441	20063	3.6	0.097	100%	100%	99%	SETD5, CHD8, SYNE1, MED13L
Feature	compositionally biased region:Poly-Glu	**3**	6	51	531	20063	2.2	0.383	100%	100%	99%	GIGYF1, CACNA1C, ILF2

DAVID tool (Database for Annotation, Visualization and Integrated Discovery) was used to analyze the genes from Supplementary Table S1, and to identify enriched biological themes, including molecular function (MF), cellular component (CC), and biological process (BP).

Supplementary Table S3 Comparison of Publications on ASDs and microRNAs (Human based) [77–91]

Study (Adult or Pediatric)	Type of Specimen	Participants (Affected/Controls)	Type of miRNA Experiment	Number of miRNAs tested /expressed	Final Results Up-regulated miRs	Final Results Down-regulated miRs	Validation	Ref
Children	Fetal brain Tissue	5, 6	miR expression, qPCR	1	miR-146a	None	Yes, H9 hNSC cell overexpression	78
Assume Adult	Brain Tissue	12, 12	miR-seq, qPCR	Sequencing	miR-142-5p, miR-142-3p, miR-21, miR-451a, miR-144-3p	None	qPCR	79
Children and Adults	Brain Tissue	10, 8	Affymetrix miR 3.0 array	1733	miR-4753-5p, miR-664-3p, miR-4709-3p	miR-1, miR-297, miR-4742-3p		80
Twins – Age not mentioned	Blood Lymphocytes	14 total; 5 autistic twin pairs - some with normal sibling	Custom miR array, qPCR	1,237	miR-29b, miR-103	miR-219-5p, miR-139-5p	qPCR	81
Assume Children	Lymphoblastoid cells	20, 22	Illumina miR array	48,000	miR-486, miR-181c	None	qPCR	82
Children	Lymphoblastoid cells	6, ?	uParaflo miR array (LC Sciences)	470	miR-23a, miR-23b, miR-132, miR-146a, miR-146b, miR-663	miR-92, miR-320, miR-363	qPCR in mouse brain	83
Children	Saliva	187, 194	miR-seq	527	miR-665, miR-4705, miR-620, miR-1277-5p	miR-148-5p, miR-151a-3p, miR-125b-2-3p	test set	84

Children	Saliva	24, 21	miR-seq	246	miR-628-5p, miR-127-3p, miR-335-3p, miR-2467-5p, miR-28-5p, miR-191-5p, miR-3529-5p, miR-218-5p, let-7-5p, miR-140-3p	miR-27a-3p, miR-30e-5p, miR-23-3p, miR-32-5p	statistical model	85
Children	Serum	30, 30	miR step-loop qPCR	2	None	miR-3135a, miR-328-3p	No	86
Children	Serum	55, 55	miR array, qPCR	125	miR-101-3p, miR-106b-5p, miR-130a-3p, miR-195-5p, miR-19b-3p	miR-151a-3p, 181b-5p, miR-320a, miR-328, miR-433, miR-489, miR-572, miR-663a	qPCR	77
Assume Adult	Serum	18, 20	miR array	Unknown	557, 486-3p	None	qPCR	87
Children	Serum	30, 30	qPCR	42	miR-619-5p, miR-664a-3p, miR-365a-3p	miR-424-5p, miR-197-5p, miR-328-3p, miR-500a-5p, miR-3135a,	qPCR	88
Children	Serum	20,23	Agilent miR array	77	miR-486-3p, miR-557	None	qPCR	89
Children	Whole Blood	7, 4	miR step-loop qPCR	26	miR-34c-5p, miR-92a-2-5p, miR-145-5p, miR-199a-5p	miR-27a-3p, miR-19-b-1-5p, miR-193a-5p	No	90
Children	Whole Blood	5, 5 then 15, 15	RiboArray MiDETECT	2,578	miR-34b	let-7a, let-7d, miR-103a, miR-1228	qPCR	91

Supplementary Figure S1: Heatmap showing differential expression of miRs in ASD from various sample types (brain, blood lyphocytes, LCLs, serum and saliva). [77–91]

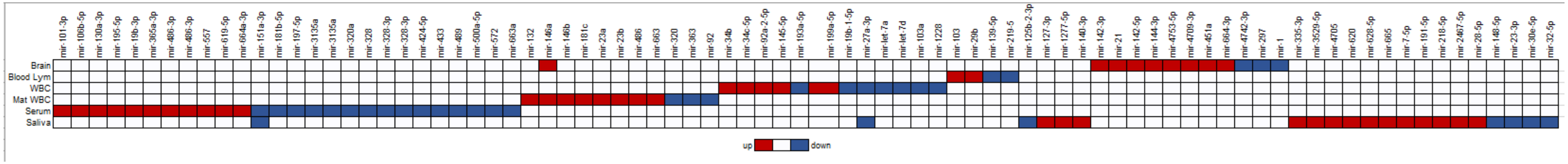

Supplement: Supplementary file 1 [file jpm-11-00848-s001.zip › jpm-1322197-supplementary.pdf]
